# Ion Mobility Spectrometry-Mass Spectrometry of Intrinsically Unfolded Proteins: Trying to Put Order into Disorder

**DOI:** 10.2174/1573411011309020004

**Published:** 2013-04

**Authors:** T. W Knapman, N. M Valette, S. L Warriner, A. E Ashcroft

**Affiliations:** 1 Astbury Centre for Structural Molecular Biology, Faculty of Mathematics and Physical Sciences, University of Leeds, Leeds, LS2 9JT, UK; 2 Astbury Centre for Structural Molecular Biology, Faculty of Biological Sciences, University of Leeds, Leeds, LS2 9JT, UK

**Keywords:** Collision cross-sectional areas, cytochrome c, electrospray ionisation, intrinsically disordered proteins, ion mobility spectrometry, mass spectrometry, osteocalcin.

## Abstract

Intrinsically disordered proteins do not adopt well-defined native structures and therefore present an intriguing challenge in terms of structural elucidation as they are relatively inaccessible to traditional approaches such as NMR and X-ray crystallography. Many members of this important group of proteins have a distinct biological function and frequently undergo a conformational change on binding to their physiological targets which can in turn modulate their function. Furthermore, many intrinsically unstructured proteins are associated with a wide range of major diseases including cancer and amyloid-related disorders. Here, electrospray ionisation-ion mobility spectrometry-mass spectrometry (ESI-IMS-MS) has been used to probe the conformational characteristics of two intrinsically disordered proteins: *apo*-cytochrome c and *apo*-osteocalcin. Both proteins are structured in their *holo*-states when bound to their respective substrates, but disordered in their *apo*-states. Here, the conformational properties of the *holo*- and the *apo*-protein forms for both species have been analysed and their mass spectral data and ion mobility spectrometry-derived collision cross-sectional areas, indicative of their physical size, compared to study the relationship between substrate binding and tertiary structure. In both cases, the intrinsically unstructured *apo*-states populated multiple conformations with larger cross-sectional areas than their *holo*-analogues, suggesting that intrinsic disorder in proteins does not preclude the formation of preferred conformations. Additionally, analysis of truncated analogues of osteocalcin has located the region of the protein responsible for the conformational changes detected upon metal cation binding. Together, the data illustrate the scope and utility of ESI-IMS-MS for studying the characteristics and properties of intrinsically disordered proteins whose analysis by other techniques is limited.

## INTRODUCTION

To gain an understanding of biological processes *in vivo* it is of paramount importance to be able to characterise the tertiary and quaternary structures of biomolecules and biomolecular complexes, to interpret the interactions required to maintain these assemblies, and to determine how these species carry out their unique functions: an edict which is the basis of structural proteomics [1]. A significantly large group of proteins, however, do not adopt a well-defined native structure and therefore present an intriguing challenge in terms of structural elucidation [2, 3]. Despite the well-accepted sequence-structure-function concept [4-6], many of these intrinsically disordered proteins have a distinct biological function [5, 7, 8], and frequently undergo a folding event to form well-defined tertiary structure on binding to their physiological targets [7]. Indeed, between 30 and 50% of proteins in eukaryotic cells are thought to lack a unique three-dimensional structure although retaining biological activity [3, 8, 9]. In terms of health and medicine, intrinsically disordered proteins have been linked with many major diseases [9-11]. For example, 79% of oncoproteins have regions of disorder of 30 residues or longer [9], and amyloid-related disorders such as Parkinson’s [12, 13] and prion diseases [9, 13] are associated with the self-aggregation of intrinsically unstructured proteins or peptides. 

Traditionally, studies of protein tertiary structure have been carried out using nuclear magnetic resonance (NMR) spectroscopy on solution samples or by X-ray diffraction of protein crystals, the data from the latter being readily convertable into high resolution atomistic structures [14, 15]. While producing some remarkable results, these techniques frequently encounter common obstacles in the form of speed of analysis or quantity of material consumed, in addition to more specific problems such as the inability to analyse large complexes or to distinguish between co-populated conformeric forms within a protein sample (NMR), or difficulties in producing high quality crystals (X-ray crystallography). A particularly challenging aspect of intrinsically disordered protein characterisation is that the unstructured, dynamic sequences of these species lead to broadened peaks in NMR spectroscopy and make their crystallisation for X-ray diffraction studies impossible.

Over the past two decades electrospray ionisation - mass spectrometry (ESI-MS) has become widely accepted as an additional technique in the field of biomolecular structural elucidation. The advantages of ESI-MS are that only low picomolar quantities of material are required, non-covalent interactions (and hence biomolecular complexes) can be maintained intact during the gas-phase analysis [16, 17], and reactions can be monitored in real-time [18-20]. ESI-MS has also been used as a probe of a protein’s conformational state. For example, individual components within a conformational ensemble can be identified via deconvolution of the ESI-MS charge state distribution [21, 22] and, additionally, protein conformation and dynamics can be probed by monitoring the rate at which backbone amide hydrogen atoms exchange with deuterons [23].

More recently, ESI-MS coupled to the shape- and charge-separation technique ion mobility spectrometry (ESI-IMS-MS), has emerged as a unique and powerful tool for biomolecular analyses, with the capability of analysing individual species within heterogeneous ensembles to provide both mass and shape (*via *collision cross-sectional area, Ω) information on all species in a single, rapid, experiment [24-33]. In particular, the relatively new technique of travelling wave IMS-MS [34, 35] has shown much potential in this area. The collision cross-sectional areas measured by ESI-IMS-MS can provide a good indication of whether a protein is folded in a native-like conformation, partially folded, or unfolded. Furthermore, if the protein co-populates several different conformers, these populations can be separated and their relative intensities estimated, as ions arising from different conformations are separable by this technique if their cross-sectional areas are sufficiently different. The more compact ions with smaller cross-sectional areas travel through the gas-filled IMS cell faster (i.e., with shorter drift times) than their extended counterparts as they experience fewer collisions with the IMS gas [27, 28, 32, 33, 36]. In the case of macromolecular protein complexes, the measurement of a cross-sectional area often requires molecular modelling to compare the Ω value obtained with the cross-sectional areas calculated for potential structures [25, 29, 37-39]. 

**Fig. (1):**
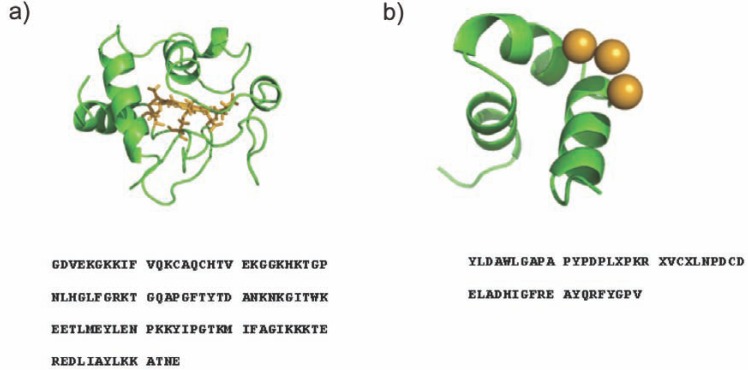
Protein sequences and PDB images of **a**) equine *holo*-cytochrome c (PDB 1GIW) [61] (the protein is shown in ribbon form and the haem ligand is shown in stick form); **b**) bovine *holo*-osteocalcin (PDB 1Q8H; porcine osteocalcin_17-49_) [54] (the three Ca^2+^ ions are shown as spheres; residue X = 4-carboxyglutamic acid).

Thus ESI-IMS-MS has the potential to provide information about the conformational properties of intrinsically disordered proteins that may be indicative of their structural preference by separation of any co-populated conformational families. Here, ESI-IMS-MS has been used to investigate the conformational characteristics of two intrinsically disordered proteins: *apo*-cytochrome c and *apo*-osteocalcin (Fig. **1**), both of which are listed in the Disprot database of intrinsically disordered proteins [40]. Cytochrome c was chosen as the properties of both the *holo*- (haem-bound) and *apo*-protein forms have been studied by other techniques and hence this is a suitable model with which to evaluate the general utility of ESI-IMS-MS for the analysis of disordered proteins. Osteocalcin is much less-well understood and so provides a real challenge to the technology. For both protein systems, the conformational properties of the *holo*- and the *apo*-protein forms have been investigated and their mass spectral data and collision cross-sectional area measurements compared. In the case of osteocalcin, truncated analogues have been generated and analysed to locate the regions of this protein responsible for the conformational changes detected upon metal cation ligand binding. The data illustrate the scope and utility of ESI-IMS-MS in this challenging field of biomolecular analysis.

## MATERIALS & METHODS

### Protein Preparation

*apo*-Cytochrome c was prepared and purified from equine *holo-*cytochrome c (Sigma Aldrich, Gillingham, Dorset, UK) by a variation of the method of Paul [41], as described by Fisher *et al*. [42]).

Bovine *apo*-osteocalcin was purified directly from bone tissue using a variation of the Gundberg protocol [43]. In short, bone fragments were pulverised to a fine powder using a Spex 6770 cryomill (SPEX CertiPrep UK, Stanmore, UK). The powdered extract was stirred for 2 h in protease inhibitor cocktail (20 mM tris-HCl containing 10 mM benzamidine and 100 µM PMSF) and subsequently centrifuged (2000 g, 20 min). Osteocalcin was extracted from its complex with hydroxyapotite by suspending the supernatant in the above inhibitor cocktail containing 250 mM ETDA for 16 h. The extract was centrifuged and dialysed against inhibitor cocktail (2 days) and water (4 days) to remove the ETDA‐hydroxyapotite complex. Extracted osteocalcin was purified by size exclusion chromatography (Superdex 200 25 mL, GE Healthcare, Buckinghamshire, UK) with 0.05 M ammonium formate as eluent, followed by ion exchange chromatography (Hitrap DEAE 5 mL, GE Healthcare, Buckinghamshire, UK) eluting with formic acid (0.07 M to 0.7 M) over 15 minutes. The purity was confirmed by SDS-PAGE. 

To generate the calcium-bound protein, *apo*-osteocalcin was mixed with calcium acetate at a protein:salt molar ratio of 1:10 which produced the *holo*-protein with three calcium ions bound. Similarly, the effects of divalent Zn^2+^ and monovalent Cu^+ ^ions were studied using zinc acetate and copper (I) acetate, respectively. 

Limited proteolysis of *holo*-osteocalcin was achieved with non-TPCK treated bovine trypsin (i.e., trypsin plus chymotrypsin; Sigma Aldrich, Gillingam, Dorset, UK) at an enzyme:substrate ratio of 1:300. The enzyme (5 mgmL^-1^ aq., 5 μL) was added to a solution of protein (24 pmolµl^-1^ aq., 100 μL) pre-mixed with ammonium acetate (50 mM aq., 6 μL).

Synthetic osteocalcin_16-49_ was prepared using Fmoc solid phase peptide synthesis (SPPS) on pre-loaded Fmoc-Leu-Wang resin. All SPPS reagents were obtained from Novabiochem (Merck Chemicals Ltd, Darmstadt, Germany) with the exception of Fmoc-4-carboxyglutamic acid (BAChem, St Helens, UK). Disulphide bond formation was achieved via slow air oxidation, as described previously [44], and confirmed by ESI-MS analysis.

### ESI-(IMS)-MS

ESI‐(IMS)-MS was performed on a Synapt HDMS (Micromass UK Ltd., Manchester, UK) orthogonal acceleration (oa) quadrupole - time-of-flight (ToF) mass spectrometer with a travelling wave IMS device situated between the two analysers, together with MS/MS facilities both before (trap region) and after (transfer region) the IMS drift cell [35]. Samples were infused into the mass spectrometer using an automated nanoESI inlet (Triversa NanoMate, Advion Biosciences Inc., Ithaca, NY, USA) with a capillary voltage of 1.6-2.0 kV. Proteins were analysed at a concentration of 13 pmolM^-1^ in 20 mM ammonium acetate buffer under native-like conditions (pH 7), and at pH 4 and pH 2 via addition of HCl.

For ESI‐MS analyses the quadrupole analyser was operated in RF-only mode and the ions *m/z *analysed by the ToF analyser; for ESI‐IMS‐MS analyses the quadrupole analyser was operated in RF-only mode and the ions separated by the IMS device before being *m/z *analysed by the ToF analyser. In the latter case, ions were accumulated in the trap ion guide and released over a period of 100 µs into the IMS ion guide. After mobility separation, temporally-resolved spectra were recorded through synchronisation of the gated release of ions for mobility separation and the oa-ToF acquisitions. Each mobility experiment subsequently consisted of 200 sequential oa-ToF spectra. 

The instrumental settings were optimised as follows: sample cone 70 V, backing pressure 3.0 mBar, IMS wave speed 250 ms^-1^, IMS wave height 7-17 V, ToF pusher interval 60 µsec, duty cycle 12 ms. The IMS nitrogen gas pressure was 5.38 ×10^‐1^ mbar and the argon gas pressure in the trap and transfer ion guides was 4.00 ×10^‐2^ mbar. The ToF pressure was 2.53 ×10^-6^ mbar. Data were processed using the MassLynx suite of programs (v. 4.1, Micromass UK Ltd., Manchester, UK). 

Mass (*m/z*) calibration was achieved on a separate infusion of caesium iodide (2 mgmL^-1^, 1:1 *v/v* H_2_O:MeOH). Calibration of the IMS device for determining collision cross‐sectional areas from drift time measurements was performed using multiply charged (M+nH)^n+^ ions from horse heart myoglobin (Sigma Aldrich, Gillingham, Dorset, UK) analysed at a concentration of 20 µM in 1:1 *v/v* 5 mM ammonium acetate: 5 mM ammonium formate at pH 2. Drift times were subjected to correction for mass‐dependent and mass‐independent flight times (the transfer times between the end of the TWIMS cell and the detector) according to equation 1 [25, 45]:

(equation 1): *t_D_´ (ms)* = *t_D_ (ms)* – *[K_EDC_ x
(m/z_ion_)^0.5^ x 1/1000 (ms)]* – *[0.9 (ms)]*

where t_D_ is the measured drift time in ms, t_D_’ is the corrected drift time in ms, *m/z*_ion_ is the mass-to‐charge ratio of the analyte ion, and K_EDC_ is a constant associated with the duty cycle of the instrument‐denoted the ‘EDC (enhanced duty cycle) delay coefficient’. K_EDC_ can be found within the software parameters: its value varies with the duty cycle of the instrument, with a value of 1.47 for a duty cycle of 12 ms. The final term of equation 1 is the mass independent transfer time (0.9 ms). 

A calibration curve was constructed according to a charge and reduced mass‐independent relationship shown in equation 2:

(equation 2) $$\Omega \left({Å}^{2}\right)=A\times {\left({t^{'}}_{D}\right)}^{B}\times \mathrm{ze}\times \sqrt{\frac{1}{m_{\mathrm{ion}}}+\frac{1}{m_{\mathrm{gas}}}}$$

where Ω is the literature-reported cross-sectional area of each calibrant obtained directly from conventional, drift tube IMS-MS experiments [46], *z*e is the charge on each ion, and the square root term is the reduced mass between the ion and the buffer gas (nitrogen). Constants A and B were subsequently derived from each calibration plot and used to calculate cross-sectional areas of unknown species from corrected drift time measurements extracted for specific *m/z* values from the data file [25, 28].

## RESULTS & DISCUSSION

### Cytochrome c 

Cytochrome c is a 104 residue, haem-bound protein located in the inter-membrane space of mitochondria. In its native state, the protein is folded into a compact, globular conformation with a single haem group covalently bound by a thioether bond to each of the two cysteine residues, Cys14 and Cys17, within the protein’s sequence (Fig. **1a**). The 3D-structure of *holo*-cytochrome c has been shown to be dependent on the presence of the haem group, as removal of this ligand results in the amino acid chain of the protein becoming significantly disordered, regardless of the solution conditions [42, 47]. 

The folded and acid-unfolded states of *holo*-cytochrome c (with the covalently bound haem group in place) have been studied by various groups using ESI-MS and ESI-IMS-MS and are generally characterised by the presence of several sets of conformers that span multiple charge states [28, 36, 48, 49]. Structural studies of the *apo*-protein (a reduced form with the haem group removed) by circular dichroism suggested that the conformations adopted by *apo-*cytochrome c are identical to those of the acid-unfolded *holo*-protein [47], indicating that the *apo-*protein is disordered without the haem interactions to anchor the protein structure in a similar fashion to a disulphide bond. 

An ESI-MS study of *apo*-cytochrome c elsewhere suggested that the protein exhibits some structural preference under native-like conditions by identifying two species [50]. This behaviour was observed by hydrogen/deuterium exchange (HDX) which gave rise to two separate ensembles exhibiting different degrees of deuterium incorporation. One ensemble showed high levels of isotope incorporation, suggesting an extended, unfolded structure, while the other exhibited significantly less exchange, indicative of a partially folded structure. Following proteolysis of the deuterated protein, the most structured region of the partially folded conformer was identified as residues 81-94. Two predominant entities have also been observed by HDX for thermally-unfolded conformations of the *holo*-protein, therefore suggesting that the process by which cytochrome c folds into its native structure relies on the formation of this structurally stable region of the protein [51].

**Fig. (2):**
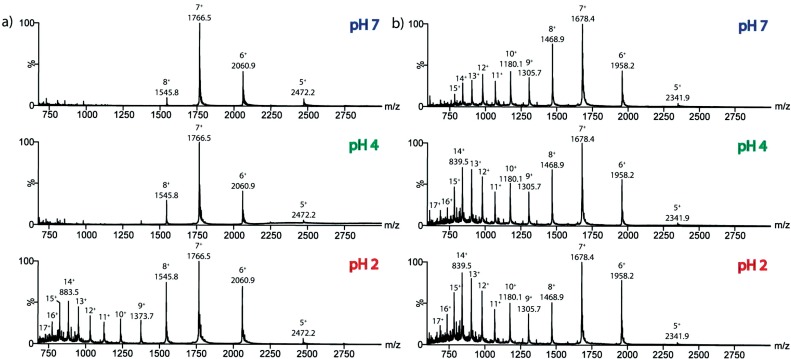
A comparison of the ESI-MS characteristics of *holo*- and *apo*-cytochrome c at different pH. ESI-IMS-MS *m/z* spectra of a) *holo*-cytochrome c (12,355 Da) and b) *apo*-cytochrome c (11,702 Da), acquired in 1:1 5mM *v/v* ammonium acetate: ammonium formate buffer at pH 7 (upper), pH 4 (middle) and pH 2 (lower).

Here, the conformational characteristics of *holo*- and *apo*-cytochrome c have been assessed and compared directly using ESI-IMS-MS. Fig. (**2**) shows mass spectra acquired at pH 7, pH 4 and pH 2 for both *holo*-cytochrome c (12,355 Da, including the haem ligand) and *apo*-cytochrome c (11,702 Da). The *holo*-protein (Fig. **2a**) was found to display ESI-MS behaviour characteristic of a native-like, folded protein. Thus, at neutral pH a relatively compact charge state distribution comprising four charge state ions (5+ to 8+) was detected, while at lower pH values, higher charge state ions were observed representing the more expanded, acid-unfolded structure of the protein (5+ to 17+), although the lower charge states arising from the folded conformers could still be observed. In contrast, a wide distribution of charge states (5+ to 17+) similar to the distribution observed for the *holo*-protein at pH 2 was observed at all three pH values for the *apo*-protein (Fig. **2b**), confirming the presence of expanded, unfolded conformeric species even at neutral pH. 

**Fig. (3):**
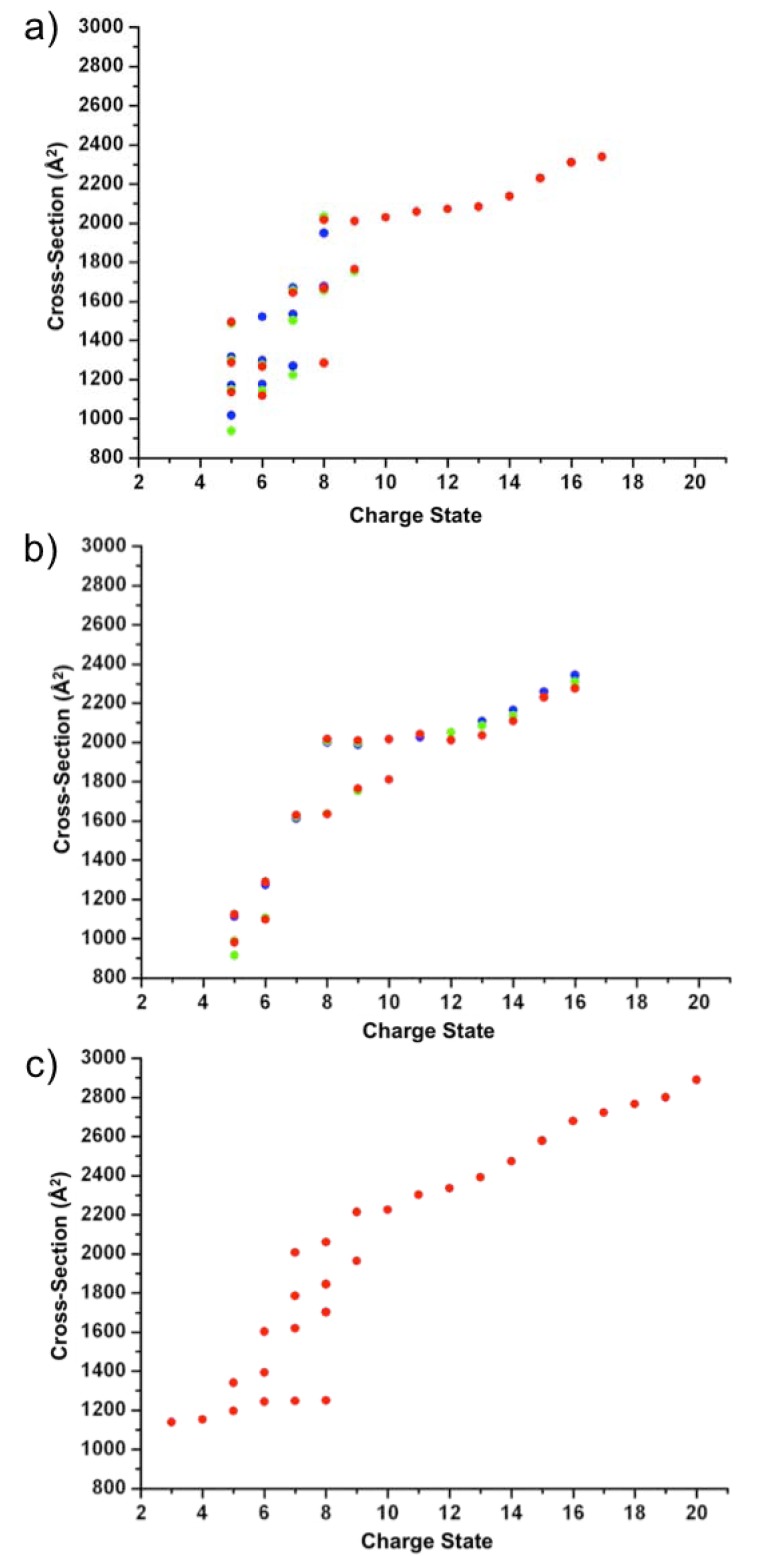
ESI-IMS-MS collision cross-sectional areas for the observed charge state ions of a) *holo*-cytochrome c and b) *apo*-cytochrome c, measured at pH 7 (blue circles), pH 4 (green circles) and pH 2 (red circles); c) Drift-tube IMS cross-sectional areas for *holo*-cytochrome c as measured by Clemmer and co-workers over a range of pH values [36].

To probe further the nature of the conformational states present, collision cross-sectional areas were measured for all of the charge states observed (some of which had contributions from multiple species) arising from both the *holo*- and *apo-*proteins at pH 7, pH 4 and pH 2. The cross-sectional areas measured for the two protein forms are shown in Fig.(**3a**,**b**) together with the drift tube IMS collision cross-sectional area data measured previously by Clemmer *et al.* [36] for comparison (Fig. **3c**). Cross‐sectional areas measured from the charge state ions of *apo*-cytochrome c at the three pH values suggest the presence of a similar number of conformers to those observed for the *holo*-protein. However, the population of the more extended species is much greater in the case of the *apo-*protein, in particular under the higher pH conditions. It is also apparent that, in the case of the *apo*-protein, the more compact conformers are populated by fewer charge states, i.e., only the 5+ and 6+ charge state ions, suggesting that the *apo*-protein is less stable in the gas phase and hence more susceptible to Coulombic unfolding. Another interesting difference between the conformers detected for the *holo*- and *apo*-proteins is that in the case of *apo*-cytochrome c, there is a distinct lack of conformers with intermediate cross-sectional areas (i.e., ~1280-1600 Å^2^), compared with the *holo*-protein for which conformers with cross-sectional areas ~1400-1600 Å^2^ are clearly detected. As a result, the *apo*-protein conformers can be grouped into two regions: the more compact conformers with cross-sectional areas ~900‐1280 Å^2^ and the more extended conformers ~1600-2300 Å^2^. This could account for the two conformeric states reported elsewhere for the *apo*‐protein in earlier HDX ESI‐MS studies [50] and highlights the fact that interfacing IMS to ESI-MS provides an extra separative dimension to these analyses which allows the intact protein to be characterised in terms of conformational states in a rapid experiment. The conformations adopted by *apo*-cytochrome c give an indication of the structural and energetic differences between the *apo*- and *holo*-states.

### Osteocalcin

Bovine osteocalcin is a small (49 residues, 5836 Da) protein with a single disulphide bond (Cys23-Cys29), three 4-carboxyglutamic acid residues (Gla18, Gla21, and Gla24) and a proline-rich region [43, 52] (Fig. **1b**). *In vivo* the protein interacts with vitamin K and hydroxyapatite to bind calcium and the resulting complex is thought to regulate bone calcification. The sequences of bovine and porcine *holo*-osteocalcin are similar and their structural characterisation has been carried out by NMR spectroscopy and X-ray crystallography [53, 54]. By NMR, both Ca^2+^ bound *holo-*proteins have been found to be composed of three helical regions with a disordered N-terminus of 15 residues that occludes a complete structural assignment; consequently only residues 16-49 of bovine *holo*-osteocalcin have been fully characterised [53]. 

Structural characterisation of *apo*-osteocalcin has thus far been fruitless, as its disordered structure causes short nucleii relaxation times and consequently poor peak resolution in NMR, and prevents crystallisation from occurring to allow X-ray diffraction data to be collected. In its *apo*-form, this intrinsically unfolded protein experiences a change to a predominantly α-helical conformation upon binding three calcium ions to the three post-translationally modified Gla residues within the protein [55]. This behaviour is well conserved between species [56, 57], despite small differences in the primary structures of the protein [58]. It has been suggested that this conformational switch causes the calcium binding sites to align within 5.4 Å of each other, which is approximately equal to the spacing of calcium ions in the crystal lattice of hydroxyapotite, thus facilitating the binding of osteocalcin to this mineral, and may subsequently play a role in bone tissue production *in vivo *[55, 57, 59].

The difficulties associated with the structural elucidation of the *apo*-form of osteocalcin suggest that the functionality of the protein may be governed by co-populated, extended conformeric species which may be amenable for resolution by ESI-IMS-MS, thus allowing insights into the protein’s conformational properties in addition to the key folding mechanism that occurs upon binding calcium ions. Hence, bovine osteocalcin was extracted and purified directly from bovine bone tissue using a method adapted from Gundberg [43] and analysed directly as the *apo*-protein by ESI-IMS-MS. To determine the effect of metal ion binding on the structure of osteocalcin, the protein was subsequently analysed in the presence of calcium acetate at a protein:salt molar ratio of 1:10, which was observed to be sufficient to observe the *holo*-protein with three Ca^2+^ ions bound while maintaining reasonable spectral quality. Previous studies of the protein have shown that the *holo*-protein is present at these relative salt concentrations [55, 60]. Additionally, the effects of other salts (divalent Zn^2+^ and monovalent Cu^+ ^ions) were investigated by using zinc acetate and copper (I) acetate in a similar manner.

**Fig. (4):**
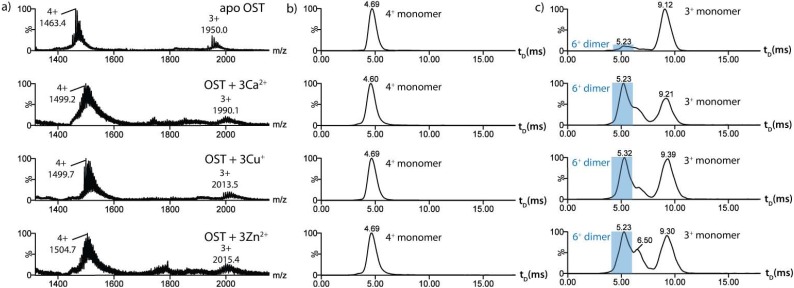
A comparison of the ESI-IMS-MS characteristics of *apo*-osteocalcin and *holo*-osteocalcin in the presence of different metal ions. a) ESI-IMS-MS spectra of *apo*-osteocalcin (apo-OST; 5836 Da), and three metal-bound *holo*-forms of osteocalcin binding three Ca^2+^, Cu^+^ and Zn^2+^ ions, respectively ((OST + 3Ca^2+^), (OST + 3Cu^+^) and (OST + 3Zn^2+^) acquired in 1:1 5mM *v/v* ammonium acetate: ammonium formate buffer at pH 7; b) IMS drift time chromatograms for the 4+ protein monomer charge state ions for the above protein analytes; c) IMS drift time chromatograms for the 3+ protein monomer charge state ions for the above protein analytes.

Mass spectra of *apo*-osteocalcin, along with three *holo*-forms (where the protein bound to three Ca^2+^, three Cu^+^ or three Zn^2+^ ions is the predominant species in each case) are shown in Fig. (**4a**). IMS drift time distributions (t_D_) for *m/z* values corresponding to the 3+ and 4+ charge states of the monomeric proteins are also presented (Fig. **4b**,**c**). In the case of the *apo*-protein, the drift time distributions for the 3+ (drift time 9.12 ms) and 4+ (drift time 4.69 ms) charge state ions are each dominated by a single peak corresponding to protein monomer, with cross-sectional areas measured as 693 and 663 Å^2^, respectively. A trace of protein dimer (6+ charge state) with a drift time of 5.23 ms and a cross-sectional area of 1054 Å^2 ^can also be identified in the drift time distribution of the 3+ charge state by inspection of the isotope ratios of this peak in its mass spectrum. In contrast to the observation of multiple conformers for *apo*-cytochrome c, the ESI-IMS-MS data for *apo*-osteocalcin show only one drift time peak for each observed charge state. There are two potential explanations for this observation: that only one predominant conformation is present in *apo*-osteocalcin, or that multiple conformers are present that are unresolved by the current technology. The second explanation is more plausible given that the peak width at half height of the 9.12 ms *apo*-protein peak corresponding to the 3+ charged state ions (Fig. **4**) is ~1.5 ms, while typical travelling wave IMS peaks for single protein conformers have been observed in the region of 0.75-0.9 ms [28]. 

Upon addition of calcium ions, the drift time profile of the 4+ charge state ions does not change but an additional species is present at 6.5 ms in the 3+ charge state profile. The mass spectral isotope pattern of this species indicates it to be a second conformer of the protein monomer. The shorter drift time of this analyte and its measured cross-sectional area of 589 Å^2^, which is significantly reduced compared with that of the *apo*-protein for this species, suggest that this peak represents a more structured *holo*-protein monomer. The observed ~17 % reduction in cross-sectional area upon calcium binding is consistent with the reported conformational change to produce a native fold in the *holo*-protein [55]. Interestingly, no such transition is observed in the 4+ charge state, potentially because the calcium-bound protein with four positive charges undergoes unfolding due to Coulombic repulsion during the ionisation stage to yield an extended conformer similar in shape/size to the *apo*-protein structure. It can also be noted that the intensity of the dimer-related peak observed in the drift time profile of the 3+ charge state ions has increased significantly on protein-metal ion binding. This is consistent with previous studies reporting that dimer formation is promoted by the binding of metal ions [57, 59]. Binding of alternative metal ions displayed a similar change in the drift time distribution, and cross-sectional areas measured for copper and zinc-bound *holo*-protein were identical to those measured for the calcium-bound *holo*-protein and *holo*-protein dimer, suggesting that the conformational change occurs regardless of the bound metal ion (Table **1**).

**Fig. (5):**
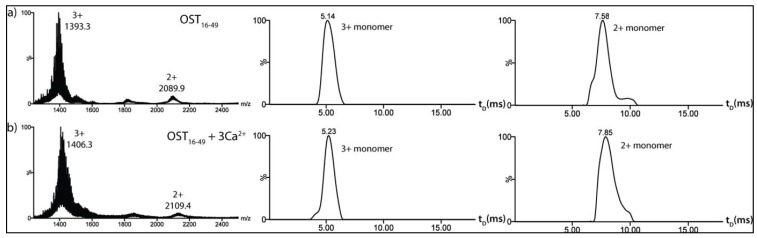
A comparison of the ESI-IMS-MS characteristics of *apo*-osteocalcin_16-49_ and *holo*-osteocalcin_16-49_. ESI-IMS-MS spectra and IMS drift time chromatograms for the 3+ and 2+ charge state ions from: a) *apo*-osteocalcin residues 16-49 (OST_16-49_), and b) *holo*-osteocalcin residues 16-49 (OST_16-49_ + 3Ca^2+^).

The apparent absence of defined structure in *apo*-osteocalcin provokes the question of how the protein performs its metal binding role given that a well-defined metal ion-binding site is unlikely to exist and, furthermore, how metal binding promotes the folding of the protein into the well-defined *holo*-structure. To study this folding process further, a truncated form of osteocalcin corresponding to the region of the protein (residues 16-49) confirmed by NMR spectroscopy to possess a well-defined structure in the calcium bound *holo*-state [53] was synthesised by solid phase peptide synthesis and analysed. Fig. (**5**) shows the ESI-IMS-MS data acquired for osteocalcin_16-49_ (4239.6 Da) alone and in the presence of Ca^2+^ ions. The *m/z* spectra of osteocalcin_16-49_, both in the presence and absence of Ca^2+^ ions, are dominated by the 3+ charge state ions accompanied by minor 2+ ions. In the case of osteocalcin_16-49_,these ions are consistent in mass with the protein monomer (Fig. **5a**). Although calcium binding to the truncated *apo*-protein is observed in the mass spectrum, inspection of the drift time distribution for osteocalcin_16-49_ with three bound Ca^2+^ ions shows very little difference to the drift time distribution observed for the truncated *apo*-protein, both consisting of a predominant peak with measured cross-sectional areas of ~420 Å^2^ and ~523 Å^2^ for the 2+ and 3+ charge states, respectively (Fig. **5a**,**b**). This suggests that no significant conformational change occurs for osteocalcin_16-49_ upon calcium ion binding, in contrast to the significant reduction in size observed in the case of intact *apo*-osteocalcin.

This observation suggests an interesting role for the disordered N-terminal region of osteocalcin in maintaining a dynamic format that can subsequently fold, or aid folding, into the correct active conformation upon calcium binding. However, the possibility that the absence of the first 15 N-terminal residues promotes the formation of an alternative conformer in both the *apo*- and *holo*-states, independent of calcium binding, must also be considered. To ascertain if this is the case, full length *holo*-osteocalcin was subjected to limited proteolysis at neutral pH to cleave the disordered N-terminus and generate a truncated form of the protein. A mixture of trypsin and low specificity chymotrypsin cleaved the protein selectively at Leu16, leaving a protein fragment of similar size (residues 17-49) to the synthetically produced truncated protein.

**Fig. (6):**
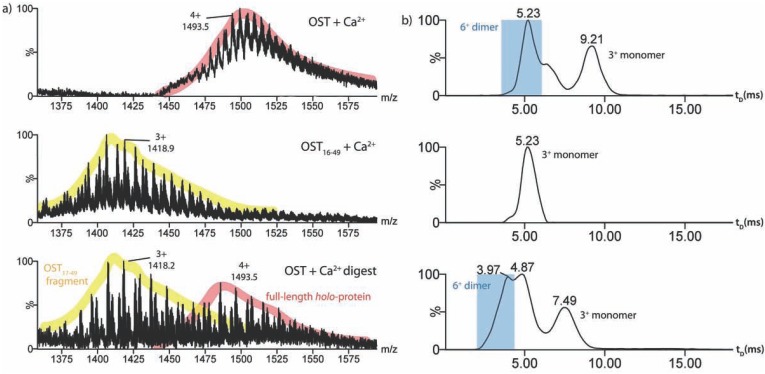
ESI-IMS-MS characteristics of full length *holo*-osteocalcin, synthetic *holo*-osteocalcin_16-49_ and enzymatically–produced *holo*-osteocalcin_17-49_. a) ESI-IMS-MS spectra showing: (upper) the 4+ monomeric charge state ions of full length *holo*-osteocalcin, (middle) the 3+ charge state ions of *holo*-osteocalcin_16-49_, each bound to Ca^2+^ ions, and (lower) the limited proteolysis mixture of the full length *holo*-protein, showing the full length (4+) and 17-49 digested (3+) species to be present simultaneously; b) IMS drift time chromatograms showing (upper) the monomeric 3+ charge state ions of the full-length *holo*-protein, (middle) the 3+ charge state ions of the synthetic truncated protein fragment (16-49) (OST_16-49_) in the presence of calcium ions, and (lower) the 3+ charge state ions of the digested 17-49 *holo*-protein fragment (OST_17-49_).

Mass spectra showing a mixture of full length *holo*-osteocalcin (4+ charge state ions) and its osteocalcin_17-49_ proteolysis fragment (3+ charge state ions) acquired during enzyme digestion were compared with spectra acquired individually for *holo*-osteocalcin and the synthetically produced, truncated osteocalcin_16-49_ in the presence of calcium ions (Fig. **6a**). Comparison of the drift time distributions for the 3+ ions of the calcium-bound proteolysis fragment, osteocalcin_17-49_, with those of the full length *holo*-protein (3+ ions) and the synthetic truncated osteocalcin_16-49_ (3+ ions) highlights the interesting properties of the intrinsically unfolded region of the *holo*-protein (residues 1-15) (Fig. **6b**). While the calcium-bound synthetic osteocalcin_16-49_ exhibited predominantly one peak with a measured cross-sectional area of ~527 Å^2^ in the drift time distribution, the osteocalcin_17-49_ fragment generated by proteolysis displayed a similar drift time distribution to that of the full length *holo*-protein, containing two peaks (drift times 4.87 and 7.49 ms, with cross-sectional areas of 508 and 631 Å^2^, respectively) corresponding to the 3+ charge state ions of two monomeric conformers. These conformers were accompanied by a peak of drift time 3.97 ms corresponding to the 6+ charge state ions of a corresponding osteocalcin_17-49_ dimer (907 Å^2^). Thus, the 3+ charge state ions of the calcium-bound and the *apo*-forms of synthetic osteocalcin_16-49_, and also the proteolysis fragment osteocalcin_17-49_, all indicate the presence of a conformeric species of cross-sectional area 508-527 Å^2 ^(i.e., a 3 % range, which is within experimental error [28, 45]). However, the proteolysis fragment osteocalcin_17-49 _also exhibited an additional conformer of longer drift time and a cross-sectional area 631 Å^2^, some 18 % larger. These data suggest that the compact conformer observed in the calcium-bound osteocalcin_16-49_ variant results from the conformer present in the *apo*-form, and not as a result of an alternative folding pathway in both *apo*- and *holo*-forms. This implies that the 15-residue N-terminus of the protein plays a key role in the transition from the intrinsically unfolded *apo*-protein to the natively folded *holo*-protein, by maintaining an unfolded state that can subsequently change conformation to the active folded form upon calcium binding. A summary of the cross-sectional areas measured for osteocalcin and its variants is presented in Table **1**. 

## CONCLUSION

ESI-IMS-MS is a valuable tool for studying conformational ensembles and processes as it enables the separation of co-populated conformers with the same *m/z* ratio but different physical size. Using this technology to study two intrinsically disordered proteins, *apo*-cytochrome c and *apo*-bovine osteocalcin, has produced interesting insights into their respective conformational behaviour patterns. The data show that it is possible to discern different conformational families even for unstructured proteins, that the conformational properties of *apo*- and *holo*-forms of the same protein can be compared and that structural events taking place during the transition from *apo*- to *holo*-protein can be monitored. For both proteins, ligand binding can be seen to allow the proteins to access more well-defined conformations, and consequently modulate biological activity.

In the case of cytochrome c, the data indicate that even at neutral pH *apo*-cytochrome co-populates a range of relatively specific conformations with cross-sectional areas ranging from 900 - 2300 Å^2^. This conformational signature is very similar to that observed for acid unfolded *holo*-cytochrome c, although at pH 4 and above the relative population of these conformations is significantly different, with *apo*-cytochrome c populating the more expanded conformational families even at neutral pH, in contrast to the structured, haem-bound *holo*-protein. 

For osteocalcin, a structural transition for *apo*-osteocalcin has been detected upon binding metal ions, with the resulting metal-bound *holo*-species having a cross-sectional area some 17 % less than its *apo*-counterpart. The *apo*- to *holo*-protein folding process is not limited to calcium ion binding, but occurs in the presence of other metal ions including copper and zinc, an observation that is in agreement with previous studies, as is the promotion of dimerisation detected upon metal binding [57, 59].

The role of specific residues in facilitating the transition from the unstructured *apo*-form to the significantly more folded *holo*-osteocalcin was also investigated. NMR studies of bovine osteocalcin have shown that residues 1-15 remain disordered in the *holo*-protein [53]. ESI-IMS-MS analysis of a synthetic, truncated form of osteocalcin without these first 15 residues (osteocalcin_16-49_) indicated that there was no detectable change in structure upon calcium binding, most likely because this truncated species adopts a more compact, structured conformation that cannot rearrange upon binding, compared with the full-length *apo*-protein. This concept was confirmed by ESI-IMS-MS analysis of a similar truncated form, osteocalcin_17-49_, which was generated by limited proteolysis of full-length *holo*-osteocalcin. Osteocalcin_17-49 _showed a drift time distribution similar to that of the full-length *holo*-protein and was observed to populate a second, significantly different conformation not observed in the case of the synthetic osteocalcin_16-49_ fragment. These data suggest that the intrinsically disordered 1-15 region of the protein plays a key role in facilitating the transition of the *apo*-protein to its active form upon calcium binding. A plausible mechanism for this process is that the disordered 1-15 region maintains an unfolded conformation in the *apo*-protein which facilitates specific calcium binding and subsequent transition to the folded state.

The data indicate the potential of ESI-IMS-MS for studying the characteristics and properties of intrinsically disordered proteins whose analysis by other techniques is not possible. A deeper understanding of the structures of these proteins will provide a greater understanding of the important biomolecular processes they govern. Of specific interest is the capability of monitoring the conformational changes which take place on ligand binding, due to the ability of this technology to separate co-populated conformers of different size/shape with the same *m/z* ratio. As intrinsically disordered proteins are over-represented in major disease pathways [11], the development of therapeutic remedies with an informative and reliable means of structural elucidation such as ESI-IMS-MS seems a future possibility.

## Figures and Tables

**Table 1.** Summary of the ESI-IMS-MS Data Obtained for the *holo*- and *apo*-osteocalcin Species Analysed.

	MW (Da)	Z (+)	*m*/z	t_D _(ms)	Ω_IMS_(Å^2^)
*apo*-OST	5850	3	1950.0	9.12	693
		4	1462.5	4.69	663
	11672	6	1950.0	5.23	1054
OST + 3Ca^2+^	5970	3	1990.1	9.21	696
				6.50	589
		4	1492.5	4.69	663
	11820	6	1990.1	5.23	1054
		7	1705.8	5.41	1250
OST + 3Cu^+^	6041	3	2013.5	9.39	696
				6.50	589
		4	1510.1	4.69	663
	11891	6	2013.5	5.32	1054
		7	1725.9	5.41	1250
OST + 3Zn^2+^	6046	3	2015.4	9.30	696
				6.50	589
		4	1511.6	4.69	663
	11896	6	2015.4	5.32	1054
		7	1727.5	5.41	1250
*apo*-OST_16-49_	4140	2	2070.0	7.58	424
		3	1380.0	5.14	523
OST_16-49_ + 3Ca^2+^	4260	2	2130.2	7.85	431
		3	1420.1	5.23	527
OST_17-49_ + 3Ca^2+^	4147	3	1376.4	7.49	631
				4.87	508
	8174	6		3.97	907
